# Exploring an Artificial Intelligence–Based, Gamified Phone App Prototype to Track and Improve Food Choices of Adolescent Girls in Vietnam: Acceptability, Usability, and Likeability Study

**DOI:** 10.2196/35197

**Published:** 2022-07-21

**Authors:** Bianca C Braga, Phuong H Nguyen, Noora-Lisa Aberman, Frank Doyle, Gloria Folson, Nga Hoang, Phuong Huynh, Bastien Koch, Peter McCloskey, Lan Tran, David Hughes, Aulo Gelli

**Affiliations:** 1 Friedman School of Nutrition Science and Policy Tufts University Boston, MA United States; 2 Poverty, Health, and Nutrition Division International Food Policy Research Institute Washington, DC United States; 3 The Global Alliance for Improved Nutrition Washington, DC United States; 4 College of Agricultural Sciences Pennsylvania State University University Park, PA United States; 5 Department of Nutrition, College of Health Sciences Noguchi Memorial Institute for Medical Research University of Ghana Legon Ghana; 6 National Institute of Nutrition Ha Noi Vietnam; 7 Hubert Department of Global Health, Rolling School of Public Health Emory University Atlanta, GA United States

**Keywords:** adolescent, dietary quality, food choice, gamification, low- and middle-income country, smartphone app, mobile phone

## Abstract

**Background:**

Adolescents’ consumption of healthy foods is suboptimal in low- and middle-income countries. Adolescents’ fondness for games and social media and the increasing access to smartphones make apps suitable for collecting dietary data and influencing their food choices. Little is known about how adolescents use phones to track and shape their food choices.

**Objective:**

This study aimed to examine the acceptability, usability, and likability of a mobile phone app prototype developed to collect dietary data using artificial intelligence–based image recognition of foods, provide feedback, and motivate users to make healthier food choices. The findings were used to improve the design of the app.

**Methods:**

A total of 4 focus group discussions (n=32 girls, aged 15-17 years) were conducted in Vietnam. Qualitative data were collected and analyzed by grouping ideas into common themes based on content analysis and ground theory.

**Results:**

Adolescents accepted most of the individual- and team-based dietary goals presented in the app prototype to help them make healthier food choices. They deemed the overall app wireframes, interface, and graphic design as acceptable, likable, and usable but suggested the following modifications: tailored feedback based on users’ medical history, anthropometric characteristics, and fitness goals; new language on dietary goals; provision of information about each of the food group dietary goals; wider camera frame to fit the whole family food tray, as meals are shared in Vietnam; possibility of digitally separating food consumption on shared meals; and more appealing graphic design, including unique badge designs for each food group. Participants also liked the app’s feedback on food choices in the form of badges, notifications, and statistics. A new version of the app was designed incorporating adolescent’s feedback to improve its acceptability, usability, and likability.

**Conclusions:**

A phone app prototype designed to track food choice and help adolescent girls from low- and middle-income countries make healthier food choices was found to be acceptable, likable, and usable. Further research is needed to examine the feasibility of using this technology at scale.

## Introduction

### Background

The consumption of healthy foods and nutrients is suboptimal worldwide, particularly in low- and middle-income countries. Although the daily intake of unhealthy foods (such as red and processed meats and sugar-sweetened beverages) exceeds the optimal consumption level, the daily intake of healthy foods (such as fruits, vegetables, grains, nuts, seeds, and fiber) is far below the recommended levels globally [1]. The importance of diets rich in fruits, vegetables, and whole grains should not be underestimated [2]. Plant-based diets prevent cancers [3] and cardiovascular diseases [4], and the consumption of fruits and vegetables has been associated with increased happiness, life satisfaction, and psychological well-being [5-7]. In contrast, sugar-sweetened beverage consumption is associated with increased adiposity, long-term weight gain, and a higher risk of diabetes [8-11]. The population of Southeast Asia has a low consumption of grains, legumes, fruits, vegetables, nuts, and seeds and a high consumption of processed meat, sugar-sweetened beverages, trans fats, and sodium [1]. Adolescents in this region have especially low consumption of fruits and vegetables [12].

Interventions intended to improve adolescents’ knowledge of nutrition and health behaviors can take advantage of new technology to self-monitor diets with feedback on performance [13,14]. Young people are early adopters of mobile phone apps, games, and social media and are highly influenced by peer norms [13,15,16]. Mobile phones and computers are now widely used by adolescents from countries with all income levels [17-19]. The implementation of nutrition interventions based on apps holds great potential to improve the dietary quality of this population, but its impact is yet to be demonstrated [17].

### Prior Work

Vietnam is a country in nutrition transition, with great changes in food supply, food prices, household expenditure, diet, and nutrition outcomes [20]. We previously reviewed the apps available in the Vietnamese market that could serve as tools for nutrition interventions [18]. Most of the apps found focused on tracking and changing food choices with the ultimate goal of losing weight [18]. Food consumption is usually tracked by searching for and selecting each food and beverage separately from a dropdown menu and their portion sizes [18]. This was considered so time consuming that even adolescents from Vietnam interested in changing their diets stopped using these apps [18]. There is a lack of free, user-friendly apps that focused on changing dietary quality [18]. We also conducted a literature review for detailed descriptions of eHealth and mobile health technologies used in nutrition interventions [18]. Intervention studies to promote healthy diets in adolescence often ignore techniques to keep participants engaged, leading to low participation and high drop-offs [18]. Adherence to these interventions is poorly reported in the literature [18].

### Research Objectives

Our goal was to develop a new app with artificial intelligence (AI) technology to recognize images of foods that can be used to track and influence the quality of food choices of adolescent girls in Vietnam using gamified nudges. Previous apps based on image recognition of foods have successfully estimated the carbohydrate content of meals with higher accuracy than individuals, predicted glycemic dynamics, and helped improve diabetes self-management [21-23]. First, we prepared a food database and an image library by (1) developing a food inventory with priority foods; (2) preparing, cooking, and taking graduated pictures of foods; and (3) annotating pictures and linking them to the food database. Second, we used annotated pictures to train a semantic segmentation model to recognize foods and estimate portion sizes (details of the AI model development are under review elsewhere). Third, we developed the *Food Recognition Assistance and Nudging Insights* (FRANI) app, which included (1) conducting formative research (2 rounds of focus group discussions [FGDs]) with users to develop its interface; (2) validating the image recognition AI technology against the 2 gold standard forms of dietary data collection: 24-hour recalls and weigh food records; and (3) integrating the validated AI technology with the user interface. The app was developed by engineers from our team.

This study aimed to examine the acceptability, usability, and likability of the FRANI prototype. Our three specific objectives were to obtain qualitative inputs on (1) the acceptability of its daily individual- and team-based dietary goals to motivate healthier food choices, (2) the usability and likability of the image capturing feature and the likability of the feedback to users (badges, notifications, and statistics), and (3) to use the inputs of participants to improve the user interface design and functionality. We hypothesized that FRANI would be acceptable, usable, and likable.

## Methods

### Study Design, Participants, and Setting

We conducted 2 rounds of FGDs with 32 adolescent girls aged 15 to 17 years in Thai Nguyen, Vietnam. The first round of FGDs was aimed at developing the FRANI prototype, and the second aim was to understand its acceptability, usability, and likability and make changes so as to improve its dietary goals, camera, and feedback (badges, notifications, and statistics). We purposely selected an urban public high school because of its good relationship with the research team. All 12th graders were excluded, because they would graduate within a few months from the recruitment period, which could undermine follow-up studies. A total of 2 classes from each of the remaining 2 grades (10th and 11th) were randomly selected, and 8 (25%) girls were randomly selected from each of the 4 classes.

They were approached by the school teachers and divided into 4 FGDs with 8 participants, each according to their school year and specialization (social science and language vs natural and biological sciences). Two of the girls initially selected did not participate because their parents thought that the study would take time and interfere with their studies. A total of 2 additional adolescents were randomly selected and replaced those who refused to participate. Randomization led to a sample of participants that varied in scholastic performance and socioeconomic characteristics as described in Table 1. The study design and reporting of findings were based on content analysis, ground theory, and the consolidated criteria for reporting qualitative research, which includes a 32-item checklist for interviews and FGDs [24].

**Table 1:** Characteristics of focus group participants (N=32).

Characteristics					Participants, n (%)
**Parents**					
	**Parent’s level of education**				
		**Mother**			
			Less than high school	9 (25)	
			High school	18 (50)	
			College	8 (22)	
			Postgraduate (master’s or PhD)	1 (3)	
		**Father**			
			Less than high school	16 (45)	
			High school	15 (41)	
			College	5 (14)	
			Postgraduate (master’s or PhD)	0 (0)	
	**Parents’ occupation**				
		**Mother**			
			Farmer	6 (16)	
			Blue-collar worker	6 (16)	
			White-collar worker	8 (22)	
			Unskilled worker	13 (37)	
			Stay-at-home parent	1 (3)	
			Other	2 (6)	
		**Father**			
			Farmer	5 (14)	
			Blue-collar worker	7 (21)	
			White-collar worker	5 (14)	
			Unskilled worker	15 (41)	
			Stay-at-home parent	0 (0)	
			Other	4 (10)	
**Household and participants**					
	**Household with assets**				
		Television		36 (100)	
		Computer		25 (69)	
		Refrigerator or freezer		36 (100)	
		Air conditioners		21 (59)	
		Washing machine		32 (88)	
		Gas cooker or stove		36 (100)	
		Water heater		33 (91)	
		Electric bicycle		21 (59)	
		Motorcycle		36 (100)	
		Car		10 (28)	
	**Participants with excellent school performance^a^**				
		Math		10 (28)	
		Physics		12 (32)	
		Chemistry		13 (36)	
		Biology		21 (59)	
		Literature		5 (13)	
		History		20 (55)	
		Geography		13 (37)	
		Foreign language		3 (7)	
		Overall		8 (23)	

^a^Excellent school performance was defined as an average grade of ≥9 out of 10.

### Research Team, Data Collection, and Analysis

A total of 2 researchers designed a semistructured questionnaire based on an analysis of the content of themes that emerged from previous FGDs [18]. The questionnaire was pilot-tested and changed according to the feedback of the pilot participants. FGDs were then facilitated in Vietnamese by a female senior researcher with a PhD degree and experience in qualitative methods from the National Institute of Nutrition of Vietnam. Moreover, 2 female researchers from the Preventive Medicine Department of the Thai Nguyen University of Pharmacy and Medicine assisted in taking notes, recording the audio, and keeping the time. The researchers did not know the participants before the FGDs. Each FGD took approximately 2 hours, and all interviews were completed in November 2020. Another researcher coded the data.

**Figure 1:**
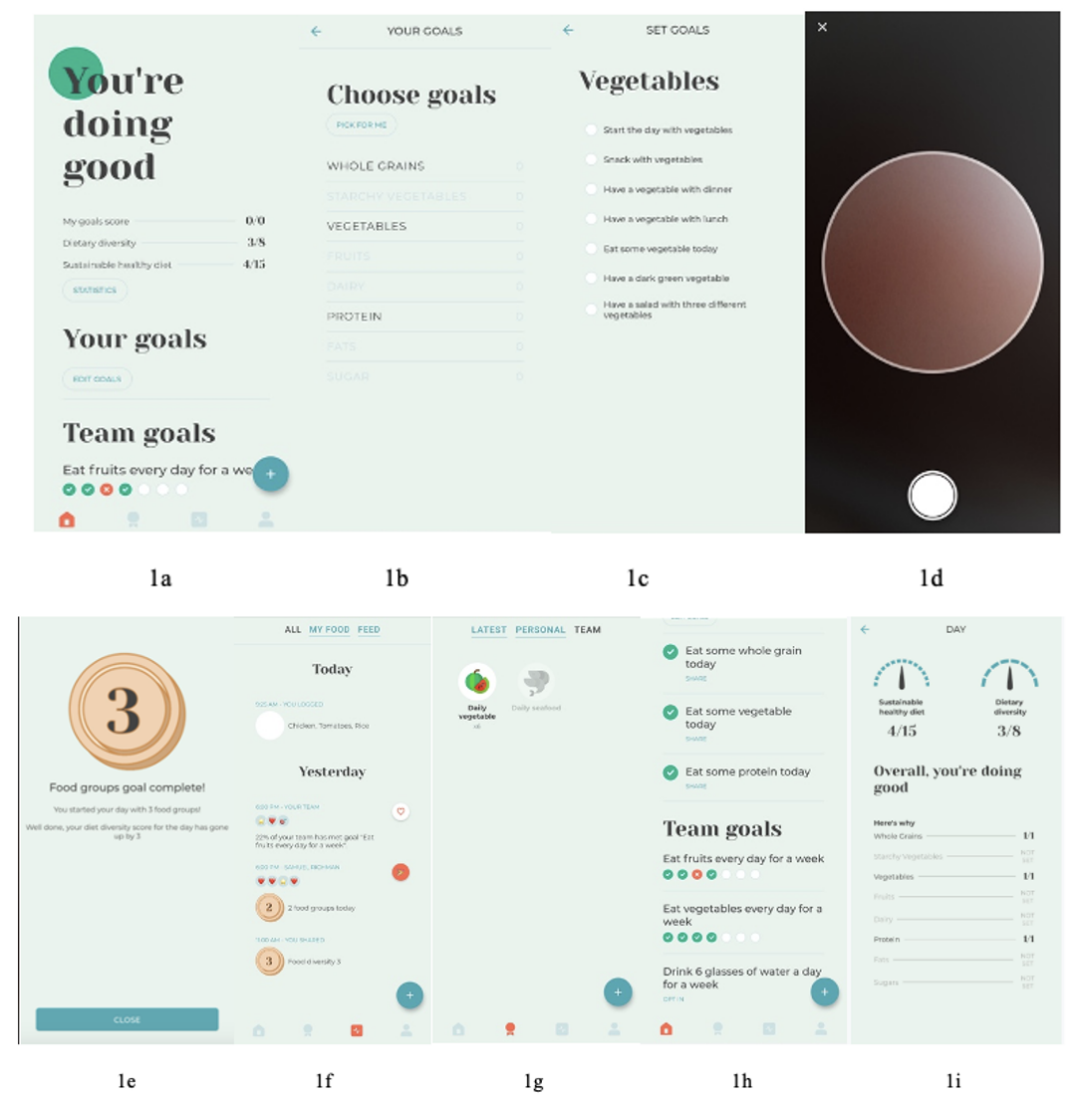
Examples of the Food Recognition Assistance and Nudging Insights wireframes presented for focus groups participants. (A) Home screen, which represents the individual- and team-based scores. The denominators were mistakenly presented in the focus group discussions (FGDs) as 8 and 15 instead of 10 and 14 signifying the food groups for the Dietary Diversity Score (DDS) and subgroups Sustainable Healthy Diet Score (SHDS), respectively. (B) All food groups for which participants could choose goals during the FGDs. (C) All possible goals for vegetables. (D) Camera frame. (E) Confirmation screen with a golden badge for completing 3 goals. (F) Activity screen in which participants can react to what other users posted. (G) Team-based badge. (H) Progress bars for team-based goals (bottom of home screen [A]). (I) Summary of daily statistics. The wireframes presented in the FGDs were written in Vietnamese.

The researchers presented the wireframes of the FRANI prototype (Figures 1A-1I, Multimedia Appendix 1) along with short instructions to guide the participants to explore the core functionality of their project-provided smartphones. Participants were guided to choose individual-based dietary goals and discussed the acceptability of each goal option. These goals for 3 food groups (vegetables, whole grains, and proteins) were selected from the lists described in Textbox 1. After selecting goals, participants took and uploaded pictures of foods using the camera tool in the FRANI prototype and then received feedback from the FRANI prototype. The facilitator asked questions on acceptability, usability, and likability, as described in Textbox 2. The FGDs were conducted in the school participants’ study.

All FGDs were tape recorded and transcribed nonverbatim. Field notes and interviewers’ observations were also incorporated into transcripts. Transcribed data from the early interviews were reviewed and discussed by the team to identify gaps in data exploration, which could be further investigated during subsequent interviews. No new information was generated in the fourth FGD, suggesting theoretical saturation. All transcripts were translated into English and randomly checked by a senior researcher who is proficient in English and Vietnamese.

Patterns within and across FGDs were reviewed and organized into the most important common themes using NVivo (version 11; QSR International) software [25]. These themes were acceptability of FRANI prototype’s individual- and team-based dietary goals; usability and likability of the FRANI prototype’s camera; likability of the feedback, including badges, notifications, and statistics; and likability of the graphic design. Transcripts did not return to the participants for comments and corrections, and interviews with the same group were not repeated. Each FGD lasted approximately 2 hours.

**List of the Food Recognition Assistance and Nudging Insights (FRANI) individual-based dietary goals that were shown to participants and discussed during the focus group discussions. The FRANI includes other food groups, but only those mentioned in this textbox were discussed with the participants.**

**Vegetables**

- Start the day with vegetables
- Snack with vegetables
- Have a vegetable with dinner
- Have a vegetable with lunch
- Eat some vegetables today
- Have a dark green vegetable
- Have a salad with 3 different vegetables

**Proteins**

- Start the day with proteins
- Snack with proteins
- Have proteins with dinner
- Have proteins with lunch
- Eat some protein today
- Vary your protein routine with beans and peas
- Have a protein food with another food group
- Bake, roast, or grill a protein food

**Whole grains**

- Start the day with whole grain
- Snack with whole grain
- Have whole grain with dinner
- Have whole grain with lunch
- Eat some whole grain today
- Try a new whole grain
- Have whole grain with another food group

**Questions asked during the focus group discussions.**

**Acceptability**

- What do you think about the dietary goals?
- What do you think about the process of setting dietary goals?
- How does having goals related to food groups help you achieve your personal goals (eg, getting in shape or being healthier)?
- How do you like having team-based goals?
- What do you think about the team-based goals?
- Would you change or improve the way the team-based goals are presented on the home screen?
- On the activity feed, how do you like seeing the combined progress of your team (“A total of 22% of your team has met the goal...”)?
- Do you like how much information can be shared on the activity feed?
- How does FRANI compare with other apps you already used to improve your habits in general, and what you eat specifically?
- How was FRANI’s information relevant for you to understand the basic principles of a healthy diet?

**Likability and usability**

- How do you like the tool for taking pictures of your meals?
- How would you change or improve the design of the confirmation screens (the immediate feedback after taking the photo)?
- How do you like the way the feedback on what you ate was provided?
- In general, how do you like the idea of receiving feedback each time right after you logged food?
- How do you like the idea of receiving medals (badges) each time you reach a goal?
- How do you like the design of the medals (badges)?
- How do you like the idea of having daily statistics about what you eat?
- How do you like the way the 3 different scores are presented on the statistics screen?
- Is the progress bar clear?
- Should the layout of that screen be changed or improved?
- Do you like using the app? Why?
- What features do you like best? Why?
- What features do you like least? Why?
- What kind of problems do you see with using the app as it’s currently designed?
- What other features do you miss in this app?
- What kind of benefits do you see resulting from using this app?
- What kind of problems do you see resulting from using this app?

### Ethics Approval

Methods and materials for the FGDs were approved by the institutional review boards of the International Food Policy Research Institute (protocol code 00007490) and Thai Nguyen National Hospital (protocol code 274/ĐĐĐ-BVTWTN). All participants assented to participate, and their parents consented to their participation. The participants were paid $50,000 Vietnamese dongs (US $2.14). All procedures were performed in accordance with the Declaration of Helsinki.

## Results

### Acceptability of the Individual-Based Goals

#### Acceptability of the Vegetable Goals

Participants generally considered the FRANI dietary goals listed in Textbox 2 achievable and suitable for a variety of people because of numerous alternatives. Respondents were confident that they could eat vegetables every day but not in all meals. All alternatives for vegetable goals are shown in Figure 1C (Multimedia Appendix 1). The goal *Eat some vegetables today* was considered easy to achieve, whereas *Eat vegetable for a snack* and *Start the day with vegetables* were considered hard to achieve. *Have a salad with 3 different types of vegetables* was seen as part of a *Western diet*, unsuited for the Vietnamese culture, because they understood a salad to be exclusively composed of leafy greens. When informed that root vegetables could be part of the salad group, they suggested the goal of having 2 vegetables, because 3 were an uncommonly high variety of vegetables for 1 meal. A group of participants suggested changing the goal *Have a salad with 3 different types of vegetables* for *eat nộm*, meaning *a mix of vegetables* in a local dialect, although the term may not be understood in other regions of Vietnam. The goal of *Having a dark green vegetable* was seen as meaningless, because they did not comprehend how the color of food could impact nutrition and had difficulty distinguishing dark green from light green vegetables. There were also concerns about intoxication from pesticide residues in the vegetables.

#### Acceptability of the Protein Goals

Respondents were unclear about what constitutes a plant-based protein. The facilitators explained that the objective of the *Vary your protein routine with beans and peas* goal was to facilitate the replacement of animal proteins with plant proteins, for example, with soybeans, peas, or tofu. Some participants held that FRANI should show examples of plant-based proteins to facilitate comprehension; others suggested changing the guidance to *Eat plant-based proteins instead of animal-based protein*. This goal and *Snack with protein* were seen as difficult to achieve, because adolescents mostly eat pastries, cakes, or candies as snacks. The healthfulness of the cooking methods of the goal *Bake, roast, or grill protein-rich foods* divided opinions. Some asked to include an explanation in FRANI about why proteins should be roasted, grilled, or baked. Others proposed boiling, air frying, and steaming, noting that well-known health influencers used these cooking methods.

#### Acceptability of the Whole-Grain Goals

Whole grains, including rice, wheat, corn, and others [26], were widely mistaken for packaged breakfast cereals:

I thought that [whole grain] was a packaged cereal.

FGD 1

Others thought whole grains were a synonym for unprocessed food or snacks:

I think that whole grains are foods that have not undergone many preparations and are pre-processed.

FGD 3

[A whole grain] is everything that is considered a snack, for example, bread, or milk.

FGD 1

Most whole-grain goals were either achievable or redundant after the leads explained their meaning. The only exception was the goal *Start the day with a whole grain*, which was perceived as difficult to achieve for those who did not eat breakfast. Participants thought that the goal *Try a new whole grain every day* required too much cooking time and creativity. To avoid misunderstanding, it was suggested that *whole grains* should be renamed.

### The Relationship Among the Dietary, Health, and Fitness Goals

Overall, participants were interested in healthy eating but felt rightly unequipped to make healthy food choices. All participants said that the FRANI should ask for weight and height, medical history, and fitness and health goals. On the basis of this information, personalized, suitable dietary goals that consider both the quality and quantity of food groups should be suggested:

There must be a complete set of information. It [FRANI] cannot help us with anything if it’s too vague. When people have filled out all the information [weight, height, medical history, and health condition], the app should make personalized suggestions so people can pay attention to what they eat.

FGD 3

Most participants would only use an app that could help them achieve fitness goals, so there was a high demand for a feature to set goals such as weight gain or loss and building muscle mass. They said that FRANI should suggest what dietary goal users should set to achieve their health and fitness goals and indicate what and how many food groups, nutrients, and calories were missing from each meal:

The app’s purpose is to promote healthy eating, [...] but I still don’t know [after choosing dietary goals on FRANI] if the way I eat is right or wrong, if it’s nutritionally inappropriate... The app has to give me advice so I can follow it.

FGD 3

Choosing goals should not be based on “users’ opinions.”

FGD 4

Other comments suggested difficulty in deciding among multiple goals, and some said that FRANI should choose their dietary goals. They also stated that FRANI should provide information about what foods are contained within each food group to avoid misunderstanding. FRANI was interpreted as an app better suited for helping with weight gain than weight loss, because receiving rewards for achieving multiple goals was associated with eating more.

### Acceptability of the Team-Based Dietary Goals and Competition

The team-based goals included *eat fruit*, *eat vegetables*, and *drink 6 glasses of water*. At the bottom of the home screen, there was a progress bar for active goals, with 1 box for each day of the week (Figure 1H, Multimedia Appendix 1). Individual members of a team would have their boxes checked if they met the goal that day. For example, if a user chose to opt in to a team-based goal of having fruits every day, they would be grouped with other users who opted in to the same goal. If the individual user achieved that team goal for that day, they would have the box checked, turning it green, or leave it red if they did not. The participants liked the idea of competing in teams. Some participants suggested adding a search engine to find users by their names, inviting contacts to join FRANI, and receiving friendship suggestions so that they could have friends as teammates.

Some participants were more excited about team-based than individual-based goals, saying that competition could trigger motivation, create connections, and foster social support. They wanted more options for team-based goals, because the team-based goals available were seen as easy to achieve:

The team-based goals are easy to achieve because these are foods that we eat every day and that are essential.

FGD 1

They would like to see team members individually ranked, with the lowest achievers at the bottom and highest achievers at the top:

Here [home screen] it must have a specific team name, and when I click on the team name, I see what goals people have achieved today and the rankings of the team members below.

FGD 3

Many claimed that underachievement would make them try harder and that they were not worried about peer pressure.

### Usability and Likability of the Image Capture Camera

Many participants said that families would support the use of FRANI as long as it helped improve the quality of their diet. However, several challenges were raised. The first perceived usability barrier of the FRANI was the size of the FRANI camera window, which is considered too small to capture images of whole family food trays (Figure 1D, Multimedia Appendix 1), because meals are typically served and eaten communally in Vietnam. Another perceived barrier to usability was the inconvenience for family members in waiting for the process of taking pictures completely before starting to eat their meals. Furthermore, separating what they eat from the rest of the family meal would be seen as disrespectful by most families, especially in the presence of older adults, house guests, and in restaurants:

If there are many people of my family [during mealtime], including the elders, it would be strange for me to separate my food from everyone else.

FGD 3

An additional usability limitation was that participants did not know how much they ate during the shared family meals:

When eating with others, we share food so it [food consumption attributed by FRANI to user] can’t be accurate. It’s complicated when many people are eating at once.

FGD 3

Similarly, after taking and uploading photos, they may consume additional servings. Thus, participants requested a wider camera frame and feature to digitally separate what users ate. Finally, participants were concerned about how much the picture could illustrate the food:

There are many things in a sandwich, but the app won’t know all the ingredients unless I take it apart.

FGD 2

They also talked about the difficulty of taking pictures of foods that come inside packages, such as potato chips. They suggested that FRANI recognizes food packages. They also wanted the ability to upload food pictures to the app after meals to avoid the inconvenience of waiting for other family members or in case there was no internet access. They would prefer to log the same picture for multiple similar meals to save time and phone memory.

### Likability of the Feedback, Including Notifications, Badges, and Statistics

Participants said they would like FRANI to give immediate feedback on the quantity and quality of foods eaten, including indications on which foods are unhealthy so as to help them make healthier choices for their next meal:

It [FRANI] should provide an immediate response so that I can learn from the previous experience for the next meal.

FGD 4

Some said they would rather receive a brief notification reminding them what they still have to eat to achieve individual-based dietary goals, than to get a daily report at night with a summary of what was eaten. They said that FRANI should also notify users about dietary goals that were not met at the end of each day and remind them to take pictures at the time of their meals. Others would like to choose the time of day to receive notifications.

Badges, or visual representations of individual- and team-based dietary goal achievements (Figure 1E, Multimedia Appendix 1), are a form of feedback that can be shared with FRANI friends on an activity screen (Figure 1F, Multimedia Appendix 1). The badge system ranges from bronze to silver to gold and streak badges praise for high dietary diversity multiple days in a row. They liked the badge system but preferred to receive only one badge per day:

I think there should only be one badge a day. This means that if you accomplished all three goals very well, you’ll get a gold badge, if you did not do too well [...] you would get a silver badge. If you didn’t accomplish any goals, you will not get a badge.

FGD 3

Participants liked the fact that friends could react (send *hearts*) when they shared their badges in the feed and agreed that comments should not be allowed to avoid cyberbullying (Figure 1F, Multimedia Appendix 1).

Participants were also presented with statistics on food intake using simple charts designed to minimize cognitive load [27]. Most participants understood the 3 different dietary scores calculated by the FRANI prototype, as explained in Multimedia Appendix 2 [28,29]. Participants liked daily statistics on dietary scores for individual goals (Figure 1I, Multimedia Appendix 1). They suggested diagrams to show if users met goals, including how much was eaten from each food item and how much they would still need to eat to achieve the goals. They would like to have a history of daily statistics permanently saved in the FRANI.

### Likability of the Graphic Design

Participants wanted the graphic design of the FRANI prototype to be more colorful and the icons to be brighter. The graphic design of the badges was considered unattractive (Figure 1E and 1G, Multimedia Appendix 1). Any food logged by participants during the FGDs showed a watermelon icon as a badge (Figure 1G, Multimedia Appendix 1); therefore, they thought the FRANI prototype did not recognize the foods correctly. This was not a recognition mistake but an unclear graphic design. They suggested that each food item should have its own differentiated badge. They also stated that graphic design should be more intuitive. The plus (*+*) icon used to take pictures on the home screen (Figure 1A, Multimedia Appendix 1) should be replaced by a camera icon.

## Discussion

### Principal Findings

This formative study investigated the acceptability, usability, and likability of an AI-based, gamified mobile phone app prototype to track and improve the nutritional quality of food choices of adolescent girls in Vietnam. The FRANI prototype was designed to self-monitor and influence food choices by considering the capabilities of and opportunities for potential users [30]. We included game elements, such as setting dietary goals and giving feedback on performance, including a badge system, notifications, and statistics, to motivate app use [31-35]. We also added elements of social media, such as sharing information with other users, a newsfeed, and the possibility to react positively to what others posted [34]. Participants of the 4 FGDs provided feedback on the app prototype, informing platform designers of their expectations [36].

### Acceptability of the Individual-Based Goals

Most participants had previously tried using various nutrition and health apps to track physical activity, diet, and health. However, they deleted these apps within a few weeks after downloading, owing to excessive battery consumption (ie, for counting steps) and time spent to complete the required tasks (ie, for logging food consumption). Adolescents preferred apps focused on behavior changes that would help them achieve immediate, potentially unhealthy fitness goals (ie, losing weight quickly) rather than focusing on health benefits (ie, improving dietary diversity). This is in line with the idea of *young invincibles* described in the literature: adolescents tend to make unhealthy food choices because of their low likelihood of developing diseases at their life stage [37]. The FRANI was carefully designed to avoid reinforcing this tendency. We opted to inspire continued use of the app by reinforcing the comprehension of the goals [34] and by increasing the saliency of healthy food choices.

**Figure 2:**
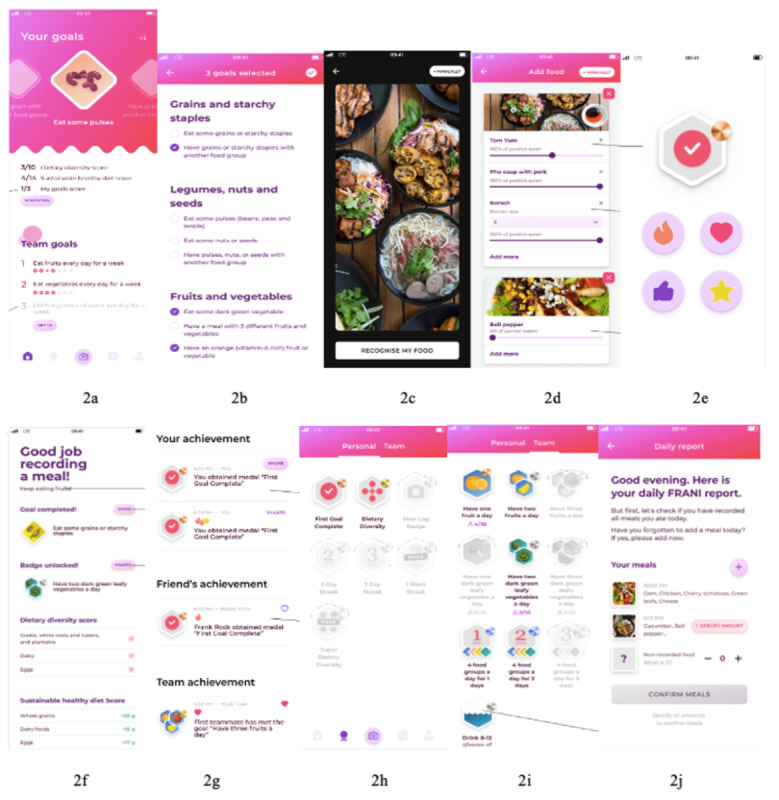
Examples of the Food Recognition Assistance and Nudging Insights (FRANI) wireframes after changes based on feedback from the focus groups participants. (A) Home screen, which represents the individual- and team-based scores with brighter colors and the right denominators for the Dietary Diversity Score (DDS) and Sustainable Healthy Diet Score (SHDS). (B) Three of the food groups for which users will be able to choose goals (they can also choose goals for dairy and meat when scrolling down). (C) Wider camera frame. (D) Slide bars to digitally indicate how much users ate from foods recognized. (E) The confirmation screen with a bronze badge. (F) Completed goals, badges, and quantities. (G) Activity screen. (H) Individual-based badges. (I) Team-based badges with 1 design for each type of food. (J) The FRANI daily report sent at night for users so they can include complete or correct information uploaded throughout the day. There are English and Vietnamese options for FRANI.

Participants accepted most of the FRANI dietary goals related to vegetable, protein, and whole-grain consumption. The dietary goals that were not understood had their language changed in the updated version of the FRANI (Figure 2, Multimedia Appendix 3). Some participants preferred FRANI to assign goals based on medical history and anthropometric characteristics, instead of choosing dietary goals themselves. This is because they rightly did not feel knowledgeable about nutrition to make choices that would optimize health and fitness. Despite their lack of confidence in setting appropriate dietary goals, FRANI was designed with the expectation that achieving the goal set would improve dietary quality, as long as the dietary goals are changed daily, and goals are achieved [18]. Furthermore, the advantages of fostering habit formation by setting specific goals [38] could be undermined by automatizing this process. Therefore, the updated version of the FRANI does not suggest dietary goals for users. This was an example of incongruency between what users said they wanted and research objectives and theory, a problem that has been previously described in the literature [39].

Following the participants’ suggestions, the updated version of FRANI’s image recognition software estimated the weight of foods and beverages and provided feedback on quantities. However, goal setting was purely qualitative because maintaining simplicity may be important to the user’s experience. The updated version of the FRANI also includes information pages with infographics and detailed explanations for food groups, goals, scores, and health implications of achieving goals. The incorporation of user goals and expectations should lead to higher motivation to use this technology and more effective future digital interventions [40,41].

### Acceptability of Team-Based Dietary Goals and Competition

Team-based challenges were widely accepted, as they were seen as a means of self-improvement driven by competition. Participants suggested a form of within-team competition: FRANI should show individual achievement information to all team members and rank them accordingly. However, the updated version does not do so, because we want to avoid excessive peer pressure, given that changes in the brain and increased testosterone during this life stage have been associated with a greater sensitivity to social evaluation and influence [42].

### Usability and Likability of the Camera

The updated version of FRANI has a wider camera frame that fits the whole family food tray and has a function to digitally separate the food portions eaten by users from the rest of the family meal. Concerning the difficulty of capturing the multiple ingredients of foods (eg, a sandwich) and foods inside packages, FRANI now also recognizes images of packages, and it is possible to manually log the ingredients that were not captured by AI technology from the picture. The manual log-in of ingredients is not only more time consuming than taking pictures of foods but also increases the overall accuracy of the app.

### Likability of the Feedback, Including Notifications, Badges, and Statistics

FRANI provides personally relevant feedback using credible nutrition information in the form of notifications, badges, and statistics. Participants demonstrated extensive interest in receiving personalized feedback from FRANI, which is important to keep users motivated and engaged [40,43], and it works better than *one size fits all* feedback [41,44]. Receiving feedback has been shown to have the potential to break undesired habits [45,46]. However, some adolescents wanted to receive negative feedback when recording unhealthy food.

In accordance with the desire of participants, the updated version of FRANI sends *just-in-time* notifications reminding users to take pictures at breakfast and lunchtime, which can lead to behavior change [47-49] and habit formation [50]. Notifications can now be turned on and off, but it is not yet possible to choose the time of the day and length of the notifications received. We chose not to send feedback disapproving unhealthy food choices or suggesting substitution for healthy foods, because it can be counterproductive and encourage unhealthy food consumption [51]. Adolescents are also particularly prone to eating disorders [52,53]; therefore, we tried to minimize the stress generated by negative feedback.

Study participants saw badges as an effective way to support behavior change, but the effects of badges, although promising, have not yet been teased out from other incentives in the nutrition behavior literature [31]. Using badges as rewards can be effective in encouraging positive responses, such as supporting desirable studying practices [33], increasing participation and engagement in schooling [54], supporting professional development [55], and stimulating voluntary contributions in web-based communities [56]. The participants were interested in self-monitoring through daily statistics and suggested that the FRANI should save historical dietary data. The updated version of the FRANI displays the dietary records.

### Likability of the Graphic Design

**Figure 3:**
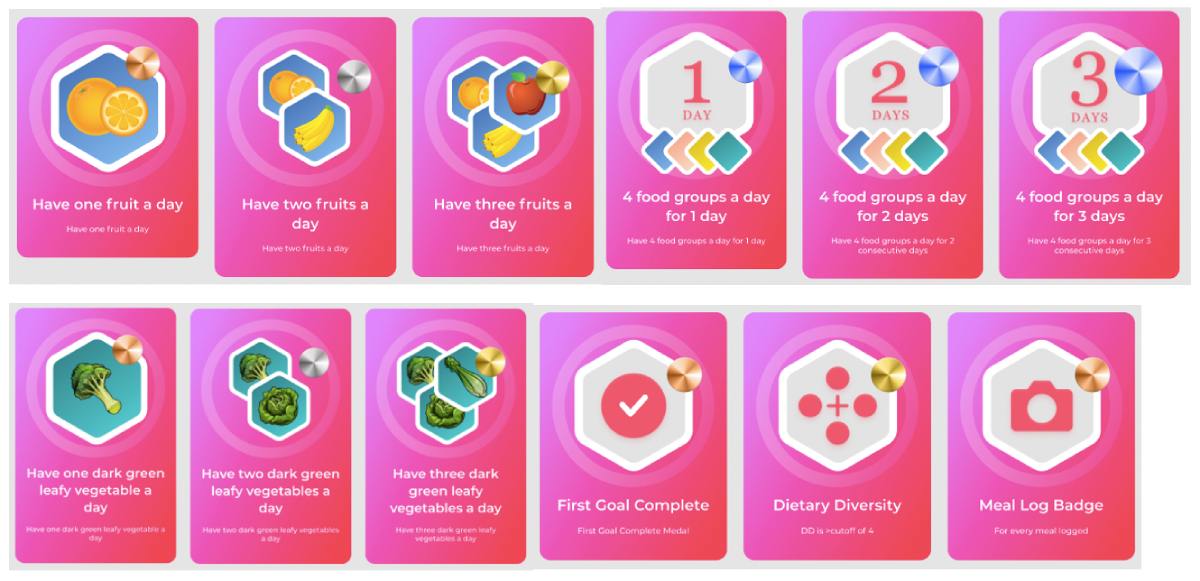
Design of new badges based on remarks from the focus group discussions. Colors are brighter than in previous badge versions, food groups have different badges, and users are leveled up from bronze to silver to gold depending on what they achieved.

In line with the ground theory, participants showed a preference for a simple interface with an appealing design [18,57-59]. The extent of the engagement benefits of eHealth and mobile health depends on the appropriate design that should be persuasive and personally relevant [60]. The updated interface was simple and visually appealing. The badges sent to praise achievements related to any food group during the FGDs had a watermelon design, which was confusing for the participants. The updated version of FRANI has badges with distinctive designs for accomplishments based on each food group (Figure 3).

### Strengths and Limitations

The main strength of this study was the in-depth qualitative assessment of the FRANI prototype. Although this assessment was based on the presentation of the prototype during the FGDs and not on real user experience, the process we describe herein can help other researchers build acceptable, usable, and likable tools for interventions. This may increase participation and adherence to future studies. As our study had a small sample size from a limited geographic location, the findings cannot be generalized to other countries, cultures, genders, and age groups.

### Conclusions

Participants accepted FRANI and deemed it usable and likable conditionally on the following modifications: (1) tailored feedback based on users’ medical history, anthropometric characteristics, and fitness goals; (2) new language on dietary goals; (3) provision of information about each of the food group’s dietary goals; (4) wider camera frame to fit the whole family food tray, as meals are shared in Vietnam; (5) possibility of digitally separating food consumption on shared meals; and (6) more appealing graphic design, including unique badge designs for each food group.

The findings on FGDs served as a guide to improve the FRANI prototype, which may also help other researchers design tools for different interventions. The acceptability, usability, and likability of the new version of FRANI, along with an assessment of the effects of using FRANI to improve food choices and dietary quality, will be quantitatively examined in a randomized controlled pilot study. To the best of our knowledge, FRANI is the first AI-based gamified self-monitoring app focused on improving the diets of adolescents in low- and middle-income countries. If successful, this tool will help to improve female adolescents’ diets and will make high-frequency data collection on food consumption possible, minimize errors, decrease long-term research costs, and help fill the gap in adolescents’ food consumption data collection [61].

## Appendix

Multimedia Appendix 1 Supplementary Figure 1.

Multimedia Appendix 2 An explanation for the dietary quality indicators present in the Food Recognition Assistance and Nudging Insights.

Multimedia Appendix 3 Supplementary Figure 2.

## Abbreviations

AI: artificial intelligence
FGD: focus group discussion
FRANI: Food Recognition Assistance and Nudging Insights

## Data Availability

The data are available on request from the authors. Thus, a formal data sharing agreement is required. The lead author has full access to the data reported in this manuscript.
