# Supplementary material for: Exploring an Artificial Intelligence–Based, Gamified Phone App Prototype to Track and Improve Food Choices of Adolescent Girls in Vietnam: Acceptability, Usability, and Likeability Study
Source: JMIR Form Res. 2022 Jul 21;6(7):e35197. doi: 10.2196/35197 (PMC9353675; doi:10.2196/35197)
Supplement: Multimedia Appendix 1 [file formative_v6i7e35197_app1.docx]

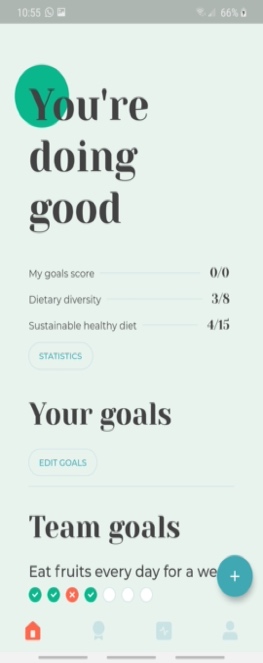

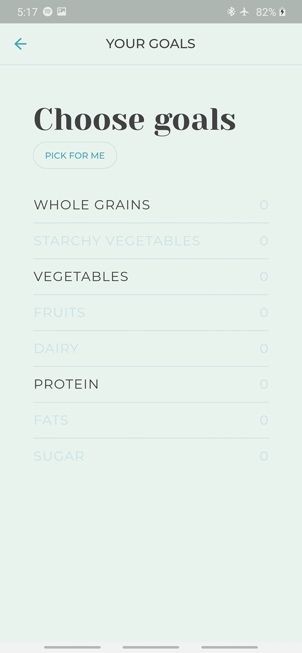

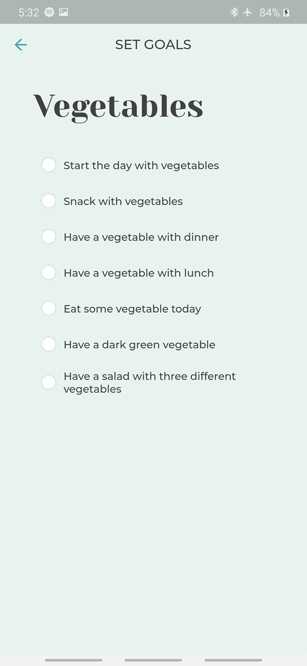

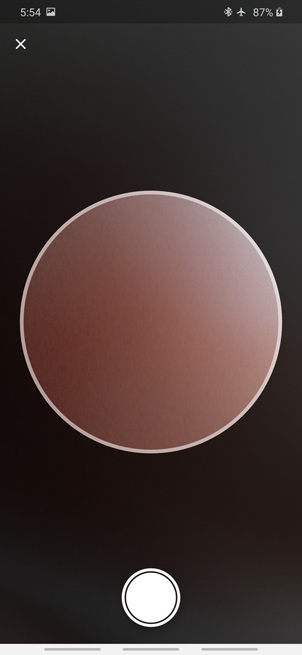


1a 1b 1c 1d


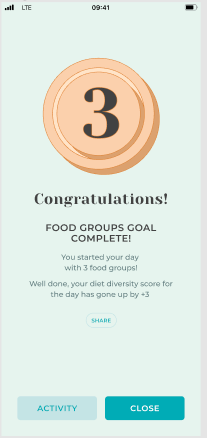

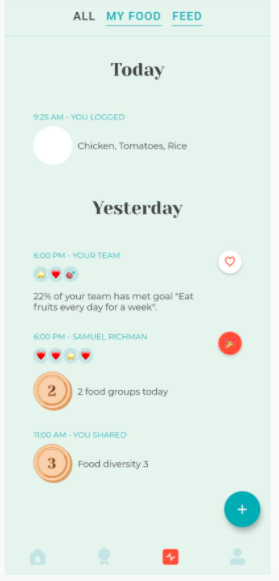

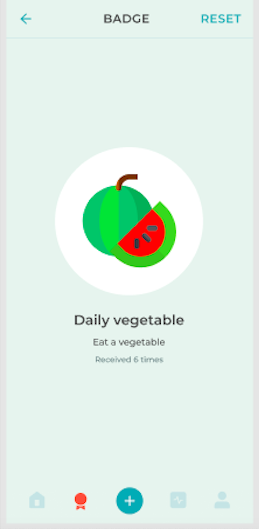

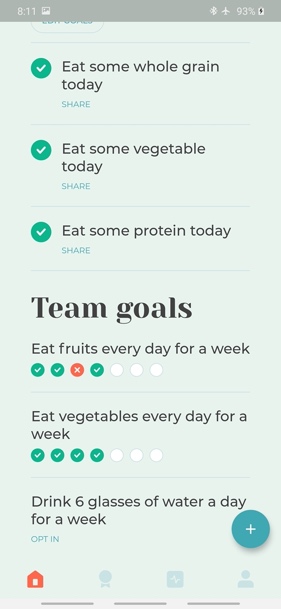

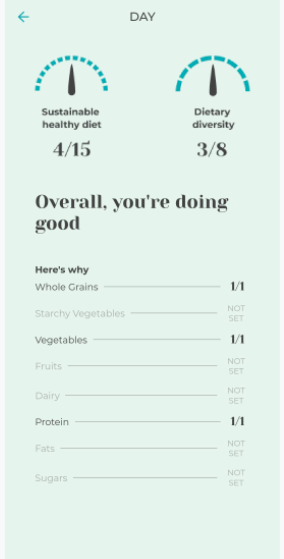

 1e 1f 1g 1h 1i

Figure 1. a-i: Examples of the FRANI prototype wireframes presented for focus groups participants. Fig. 1a home screen, which represents the individual- and team-based scores. The denominators were mistakenly presented in the FGDs as 8 and 15 instead of 10 and 14 signifying the food groups for the DDS and sub-groups for SHDS, respectively. Fig. 1b all food groups for which participants could choose goals during the FGDs. Fig. 1c shows all possible goals for vegetables. Fig. 1d. camera frame. Fig. 1e confirmation screen with a golden badge for completing three goals. Fig. 1f activity screen in which participants can react to what other users posted. Fig. 1g shows team-based badge. Fig. 1h progress bars for team-based goals (bottom of home screen 1a). Fig. 1i summary of daily statistics. The wireframes presented in the FGDs were written in Vietnamese.
