# Supplementary material for: Exploring an Artificial Intelligence–Based, Gamified Phone App Prototype to Track and Improve Food Choices of Adolescent Girls in Vietnam: Acceptability, Usability, and Likeability Study
Source: JMIR Form Res. 2022 Jul 21;6(7):e35197. doi: 10.2196/35197 (PMC9353675; doi:10.2196/35197)
Supplement: Multimedia Appendix 2 [file formative_v6i7e35197_app2.docx]

**Appendix 2: FRANI dietary scores**

FRANI uses three different scores to evaluate the healthiness of diets, the Dietary Diversity Score (DDS), the Sustainable Healthy Diet Score (SHDS), and the My Goals Score. The DDS uses a 10-food group classification from an existing indicator—the Minimum Dietary Diversity for Women (MDD-W) [60], and calculates a score based on the sum of all distinct food groups in the participants’ diets each day. The 10 food groups are grains, white roots, and tubers and plantains; pulses (beans, peas, and lentils); nuts and seeds; dairy; meet, poultry and fish; eggs; dark green vegetables; other vitamin A-rich fruits and vegetables; other vegetables; and other fruits. The SHDS counts the number of food sub-groups which consumption stayed within a desirable quantity range from the EAT-Lancet diet report. The EAT-Lancet Diet tries to balance nutrition with environmental concerns [26, 61]. The score ranges from zero to 14 per day based on 14 food sub-groups: rice, wheat, corn and other; potatoes and cassava; all vegetables; all fruits; whole milk or derivative equivalents; beef, lamb, pork; chicken, other poultry; eggs; fish; dry beans, lentils, peas; soy foods; peanuts or tree nuts; palm oil, unsaturated oils, dairy fats (including milk), lard or tallow; and all sweeteners. whole grains, tubers, and starchy vegetables, vegetables, fruits, dairy, protein sources, legumes, added fats, and added sugars. Perfect scores for the DDS and the SHDS would be given for those who eat all food groups of DDS and stayed within the desirable quantity range of all sub-groups of the SHDS on a given day. The My Goal score is the proportion of food groups eaten from the food groups chosen by FRANI users as their personal dietary goals for a given day. FRANI has three dietary quality scores to allow users to explore different aspects of diets that are complementary to each other.
