# Supplementary material for: Exploring an Artificial Intelligence–Based, Gamified Phone App Prototype to Track and Improve Food Choices of Adolescent Girls in Vietnam: Acceptability, Usability, and Likeability Study
Source: JMIR Form Res. 2022 Jul 21;6(7):e35197. doi: 10.2196/35197 (PMC9353675; doi:10.2196/35197)
Supplement: Multimedia Appendix 3 [file formative_v6i7e35197_app3.docx]

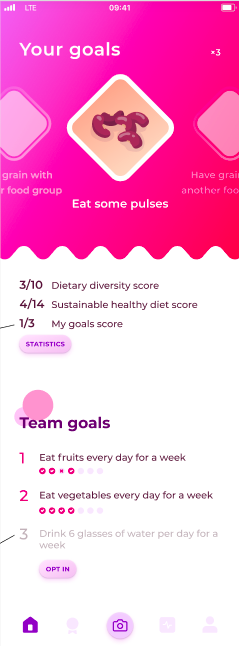


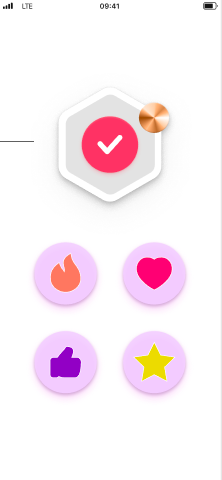

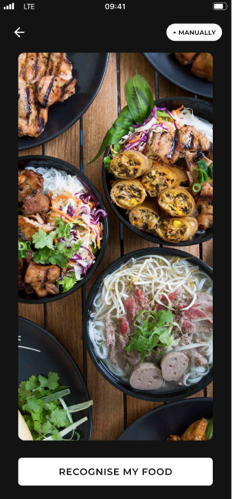

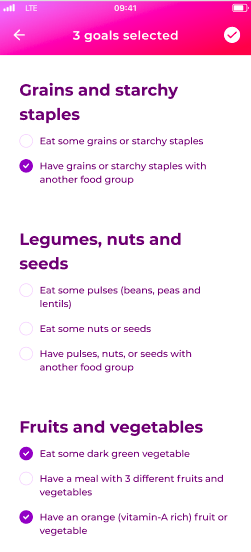

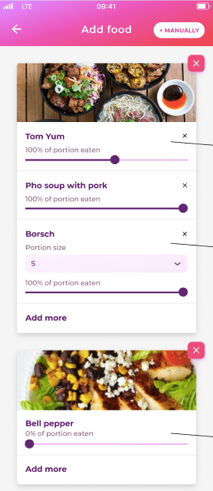


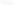

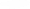

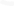

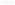

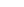

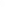

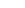

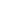

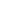

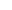

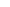


2a 2b 2c 2d 2e


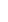

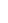

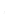

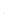

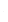

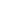

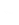

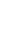

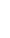

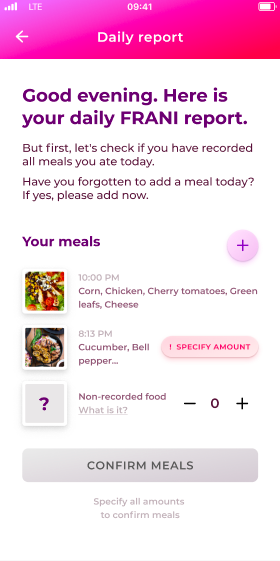

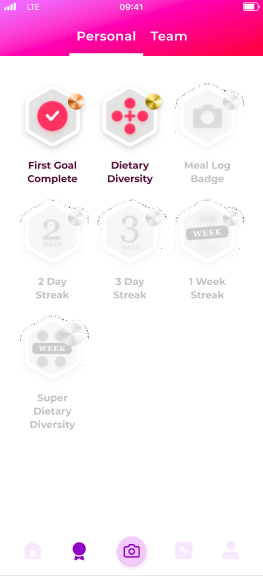

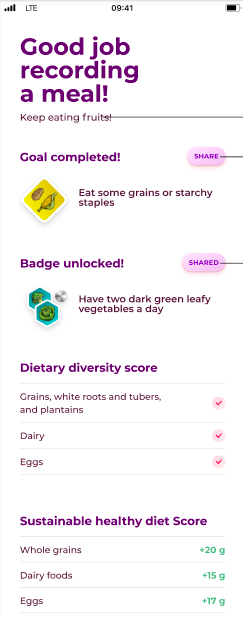

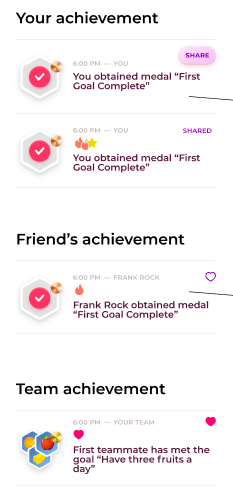

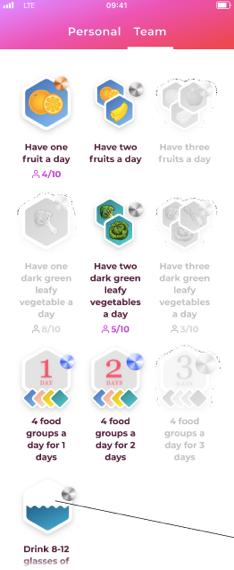


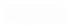

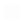

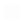

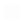

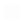

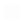

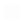

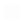

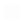

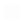

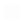

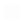

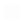

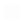

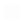

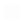

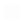

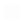

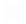

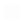

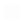

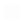

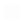

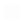

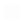

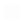

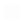

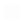

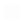

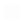

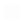

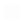

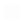

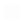

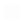

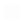

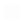

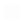

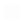

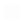

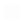

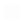

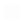

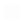

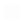

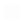

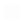

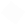

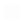

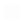

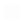

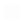

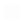

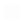

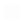

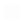

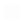

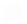

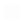

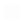

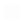

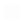

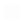

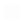


2f 2g 2h 2i 2j

**Appendix 2a-2j:** Examples of FRANI wireframes after changes based on feedback from the focus groups participants. Fig. 2a home screen, which represents the individual- and team-based scores with brighter colors and the right denominators for the DDS and SHDS. Fig. 2b shows three of the food groups for which users will be able to choose goals (they can also choose goals for dairy and meat when scrolling down). Fig. 2c wider camera frame. Fig. 2d slide bars to digitally indicate how much users ate from foods recognized. Fig. 2e shows the confirmation screen with a bronze badge. Fig. 2f summarizes completed goals, badges, and quantities. Fig. 2g activity screen. Fig. 2h individual-based badges. Fig. 2i team-based badges with one design for each type of food. Fig. 2j FRANI daily report sent at night for users so they can include, complete or correct information uploaded throughout the day. There will be English and Vietnamese options for FRANI.
